# A Case of Acute Poisoning by Arsenic, Ending in Recovery

**Published:** 1865-09

**Authors:** J. R. Lothrop


ART. IV—*A Case of Acute Poisoning by Arsenic, ending in recovery.* By J. R. Lothrop, M. D.

It will assist a clear understanding of the following details of the case, to relate the facts which led to a suspicion of poisoning by arsenic. A boy in the employ of a farmer, an inmate of his family, was known to have bought arsenic. On the morning of Wednesday, August 16th, the wife and a son of the farmer breakfasted together. Both were soon attacked with vomiting. The wife died that night with symptoms of poisoning by arsenic, the son, though made quite sick, recovered by afternoon. At noon the farmer, a physician called to attend the wife, and a little daughter whose case is related below, dined together, and were all soon after attacked with vomiting. In the case of the little girl the symptoms were serious and long continued. The others soon recovered and felt no ill effects. Upon examination Dr. Hadley found arsenic in the liver of the wife, thus establishing the fact of poisoning by arsenic.

On Wednesday, August 16th, in the evening I was called to see a case of suspected poisoning. The patient was a girl of about ten years of age. The following facts were made known to me when I first saw her. In the morning she went from her home, at her grandmother's, to her father's farm, a few miles out of the city, dined and returned in the afternoon about 5 o'clock. Soon after her return she was attacked with vomiting, which continued at short intervals. She stated that she was taken sick soon after eating her dinner and vomited several times before her return. From something which was said about arsenic having been bought the day before by a boy at the farm, the family became fearful that she was poisoned. When I first saw her she was lying upon a couch, apparently well, with no heat of skin or increase of pulse. Soon after she vomited with considerable retching and ejected a small quantity of watery liquid, appearing after it was over as comfortable as before. This was repeated in a short time, perhaps fifteen or twenty minutes. The vomiting was not preceded by much nausea. She would appear free from it till just before vomiting occurred, then she would complain of being sick and immediately vomit.

A mustard emetic had been given before I saw her, and she was allowed to drink water freely. As soon as it could be procured, the hydrated sesquioxyde of iron was freely given and frequently repeated, with a liberal allowance of water. The vomiting continued at short intervals till nearly midnight, when the intervals became longer, and towards morning it ceased, and the patient slept. At 6 A. M. Thursday, the trouble seemed over, and I began to feel that there would be no return. I then ordered powdered magnesia to be given, with the intent to clear the intestines of whatever they might contain, of iron and arsenic, and to forestall all danger of fresh absorption. Very soon after the magnesia was given vomiting began anew, and continued till the next day, Friday, when about noon it ceased entirely. As soon as the vomiting was renewed the iron was again given, and its administration kept up till noon of that day, Thursday. At that time it was discontinued, as it seemed probable that the limit of its usefulness as an antidote had been reached, and its further use was more likely to irritate the stomach than to be attended with benefit. The giving of magnesia was then resumed mainly for the purpose of acting upon the intestines to expel the iron and arsenic which might be in them. For though it might be supposed that but little iron would pass the pylosus during the continued vomiting, in fact, a considerable quantity did pass, and was afterwards expelled; and as to arsenic, though it was probably taken in solution and would not therefore, if let alone, be likely to be found undissolved in the intestines, if the iron had the power to render it insoluble it would be found there with it and be expelled at the same time. The magnesia in fine powder was given in milk, and though apparently all vomited, yet enough passed into the bowels to produce after a time, aided by an enema, a pretty free evacuation of their contents. Magnesia was given frequently till late in the evening and then discontinued.

Up to this time, late Thursday evening, with the exception stated above, vomiting had persisted at frequent intervals, the intervals being seldom over half an hour, and often but fifteen minutes. It had continued then for more than twenty-four hours. At first, though there was almost no distress, even with considerable retching, as it went on both increased, and the patient began to complain of pain and soreness, and at times there were streaks of blood in the liquid ejected. There was no tenderness upon pressure, and the abdomen was much retracted. No attempt had as yet been made to restrain the vomiting; inasmuch as it had appeared salutary, and had not been attended with so much discomfort or general disturbance as to demand interference. Now, however, the general disturbance was considerable; there was much heat of skin, a rapid and irregular pulse, having increased till it counted one hundred and twenty per minute, and in addition to the distress spoken of, the patient was excited, restless and thirsty, calling for water, and seeking relief from the heat of skin by frequent bathing. Deeming it proper to attempt to restrain the vomiting, I gave, by the mouth, an eighth of a grain of morphine, and repeated it every half hour, three or four times. The effect of this was seemingly to make the vomiting more frequent, and a failure to control the other symptoms. I then made a subcutaneous injection of morphine, using a quarter of a grain. This was followed by a decided improvement. In two hours I repeated the injection, using half a grain. After this still more improvement was manifest. The vomiting did not cease, but the intervals were greatly prolonged, the retching was much moderated, and the distress removed. After midnight, when the last injection was made, the patient slept somewhat, was much more quiet and comfortable, and the pulse became slower, being reduced to about one hundred per minute. During the forenoon of the next day, Friday, although she vomited occasionally, she had no pain, and was inclined to sleep, being drowsy and delirious. About noon, as the effect of the narcotic had worn off, I made another injection, using one-third of a grain. Soon after this was given vomiting ceased, and by evening the little patient appeared bright and natural; seemingly well. She slept well the night following, and during the next day was ready to take light food and able to run about.

Such in considerable detail is the history of a case which I watched with great interest. Evidently the quantity of arsenic absorbed was small as there was no purging, and there was no very marked prostration, nothing approximating the collapse seen in fatal cases. Yet there was enough to peril life and to create some doubt as to the result. The symptoms were most intense during the time within which severe cases end fatally, for death occurs generally in such within forty-eight, and may take place in a few hours. There was no irritation of the urinary passage or strangury, though the urine was scanty. An eruption of rather dark spots on the face was noticed, but it was not looked for on the body. The heat of skin was somewhat variable, coming in flashes, and there were slight spasmodic movements. The tongue became furred and red at the tip and sides, but not dry or fissured. The thirst was great, and not easily satisfied, as water was soon rejected. The symptoms continued through forty-eight hours. The books speak of a burning as of a consuming fire, and excruciating pain, but it is probable that an intense distress and restlessness such as we see in cholera, will more often be observed, though the act of vomiting itself may become for the time painful.

*Vomiting.*—Arsenic is said to cause vomiting by acting as an irritant to the stomach. Yet it causes it as certainly when introduced into the system in any other way. Irritation cannot be the whole of its action in producing vomiting, and in fact is probably but a small part of it. The signs of irritation are not very apparent after death. Traces of inflammation of the stomach must be very rarely observed, or in fact any traces. The softening of the mucous membrane so often mentioned is probably more due to the action of the gastric fluid, after death, as it is found in the stomach in large quantity usually in fatal cases, and the fluid vomited is often sour to the smell and probably taste, is in fact largely the gastric juice—the stomach being excited to secretion, either in consequence of prolonged vomiting, by no means uncommon, or because there is an attempt at elimination. The same may be said of the purging. The copious evacuations may be an effort to eliminate the poison, rather than a consequence of its action as an irritant. The cases in which a large quantity of arsenic is probably taken with food, and afterwards rejected by vomiting without causing any very great disturbance, hardly more than would be caused by an ordinary emetic, seem to be exceptions. If the vomiting is not merely mechanical in such cases, but is the result of absorption, how is it that they will get off with just that amount which will vomit, but not dangerously poison? Many persons partake of the same poisoned substance, yet one only dies, and the rest are not seriously affected. The difference probably is in the quantity of food taken, and the state of the arsenic. Arsenic taken unsolved into the stomach is more likely to cause serious if not fatal effects than when in solution. Some of it adheres in masses to the stomach, and is therefore not expelled with the food. The chances of absorption are much greater then, than when it is in solution and mixed with food, for only that small amount which comes in contact with the stomach walls is absorbed, an amount in many cases just sufficient to disturb the system to vomiting, and thus ensure safety. But again the quantity of food taken still further affects it, so that the less food taken the greater the danger. Something like this will account for the differences in cases where the poison has been taken under circumstances which seem to make it certain that all will be affected alike. When masses cling to the stomach absorption goes on for a time certainly, but as will be seen from what follows, we may doubt whether the stomach retains the power of absorbing a long while after vomiting sets in. If water is not absorbed there is little reason to suppose any other substance will be. An inference from this would be that the poison must do its fatal work early, and that the absorption whether sufficient in amount to destroy life or not must take place before much vomiting has occurred.

In the case described it was observed that tbe quantity vomited always exceeded the quantity taken. This was true when water was taken freely, but was much more evident when water was sparingly used. After vomiting had continued for some time and less water was taken, being purposely withheld in large quantities, from a notion, erroneous probably, that the vomiting would thereby be made less frequent, at least twice as much liquid was ejected by the stomach as was taken in. The stomach evidently secreted a part of the liquid, and this fact explains in a great measure the insatiable thirst which torments the patient. There is not only no absorption of water to satisfy the thirst, but the water of the blood is constantly drained away. Thirst is a general want, though located in or recognized by the stomach. The body calls for water when none can be supplied, hence the intense thirst, and hence too the dryness and burning of the skin which is often experienced. On this account bathing the skin is very grateful. The fact of this secretion of liquids by the stomach explains in part the enormous distention of the stomach by accumulated fluids, which is very frequently found in examinations after death from poisoning by arsenic. As the powers fail the vomiting ceases, but the secretion goes on for a time. No doubt a portion of the liquid is taken in to satisfy thirst and not rejected. We might infer then that the urine would be scanty and even suppressed, adding uræmic poisoning to the other morbific influences. Secretion, perhaps it would be better to say excretion is active by the stomach and bowels, but the functions of all other excretory organs are disturbed in consequence. The blood loses its vital properties and becomes incapable of maintaining life. All this must be understood to be in addition to or a part of the morbific influence of arsenic, whatever that is.

*Elimination.*—Probably all organs which eliminate water aid to rid the blood of the presence of arsenic. I have already suggested that the stomach and the whole intestinal tract were probably active in its excretion. But the kidneys probably effect by far the greater portion in the work of taking it from the body. During the earlier, i. e. during the first twenty-four hours, the quantity escaping in the urine is largest, but quite likely it could be detected in the urine for several days or a week. In the case related, the urine of the third twenty-four hours was found by Dr. Hadley to contain arsenic. The liver is supposed by some to aid in excretion. A large quantity is almost always found in the liver, and this we should expect independent of any excretory function in the organ. The portal blood carries the arsenic to liver soon after its absorption, and therefore with the other organs having connection with the portal system it gets an early supply of the poison. Moreover, the peculiarities of the circulation in the liver, easily account for the large proportionate quantity arrested in it. In so far as the liver has an excretory function it probably is efficient in aiding elimination, but no farther. The urine is at first quite copious, but becomes scanty or suppressed sooner or later. If water could be ingested the urine would probably continue to be secreted freely—its scantiness can readily be accounted for from the want of water in the blood. Of course if water could be absorbed nothing could be more rational than to supply it freely for the purpose, among others, of freeing the blood of the poison by the kidneys. In chronic cases elimination by the kidneys is probably the principal method of relief. Therefore depurative diuretics are highly useful.

*Treatment.*—It is obvious that the aim of all treatment is to get the arsenic from the body. Nature sets to work her agencies very early, and vomiting is one of the first methods. The emetic operation of arsenic, especially when taken in solution is often remarkably rapid. One case is known to the writer, in which the arsenic was put in the tea, and several persons of the family were attacked with vomiting before the conclusion of the meal. Spontaneous vomiting as has been made clear often prevents all future danger. Vomiting then is the first method of removal. Nor should any attempt be made to restrain it at first, though probably not much could be affected by safe doses of opium. If the arsenic has been taken in solution there is less benefit in prolonged vomiting, as far as removal from the stomach is concerned, because none is likely to remain in it, but if it has been taken in powder there is more need of it; for even after continued vomiting, after death it is found adherent to the lining membrane, especially if much has been taken. Vomiting then should be aided by liquids. As an antidote the hydrated sesquioxyde of iron may be employed. This should be given freely in large doses as often as every fifteen minutes. A large portion will be rejected almost as soon as taken, and therefore may be given for a considerable time. When we lay aside the idea of gastritis, or gastric irritation, and look upon vomiting as not only an effort to expel the contents of the stomach, but a means of elimination, we shall be less afraid of producing the morbid states which we have assumed to exist.

After vomiting has continued for some time and retching and distress become great, an attempt to relieve these by opiates seems very proper. To effect this the best method by which the action of opiates can be secured is by subcutaneous injection. It is evident none would be retained or absorbed by the stomach, and often the rectum has as little retentive or absorbing power. But the giving of opium may do harm as well as good. It will inevitably retard the process of elimination, especially by the kidneys, as well as relieve the distress. It should not therefore be given early unless the distress is great. It would be desirable to aid elimination by those agents which are thought to increase it, such for instance as iodide of potash, but as no medicine of this nature can be given on account of the vomiting, we are compelled to leave the process to the natural efforts. When vomiting ceases, and especially if there has not been much purging, a laxative is required. These measures are mainly to be kept in mind in those cases in which the quantity taken though large is not inevitably destructive. In many cases the amount absorbed is so great that no measures can afford the slightest hope of benefit, and death soon takes place. In others prompt and judicious treatment will save life.

*Antidotes.*—It is matter of doubt whether the action of iron is anything more than mechanical, and therefore like that of magnesia and other insoluble or slowly soluble powders, enveloping but not chemically changing arsenic. It probably does more than play this negative part, by rendering the arsenic insoluble and precipitating it from its solution. This is the opinion of Dr. Hadley, whose authority is entitled to great weight. But whatever part it plays it can have no action on arsenic already absorbed. The only method of getting rid of that is by elimination. The hydrated sesquioxide of iron may be very readily prepared by adding water to the tincture of the muriate of iron and precipitating by ammonia. The precipitate is to be washed till it ceases te ammoniacal. It can be thrown upon a large cotton filter and the water squeezed out. When prepared the powder may be given mixed with water. This however is only one of the methods of obtaining, and is mentioned because the materials for its preparation are more likely to be at hand. Mialhe proposes the hydrated sulphuret of iron as of superior efficacy. There is probably no special advantage to be derived from its use, and it is best to content ourselves with the readily obtained hydrated sesquioxide.

